# Progesterone receptor membrane component 1 facilitates Ca^2+^ signal amplification between endosomes and the endoplasmic reticulum

**DOI:** 10.1016/j.jbc.2023.105378

**Published:** 2023-10-20

**Authors:** Gihan S. Gunaratne, Sushil Kumar, Yaping Lin-Moshier, James T. Slama, Eugen Brailoiu, Sandip Patel, Timothy F. Walseth, Jonathan S. Marchant

**Affiliations:** 1 Department of Cell Biology, Neurobiology & Anatomy, Medical College of Wisconsin, Milwaukee, Wisconsin, USA; 2 Department of Pharmacology, University of Minnesota Medical School, Minneapolis, Minnesota, USA; 3 Department of Medicinal & Biological Chemistry, University of Toledo College of Pharmacy and Pharmaceutical Sciences, Toledo, Ohio, USA; 4 Center for Substance Abuse Research and Department of Neural Sciences, Lewis Katz School of Medicine at Temple University, Philadelphia, Pennsylvania, USA; 5 Department of Cell and Developmental Biology, University College London, London, UK

**Keywords:** calcium signaling, heme, NAADP, endosomes, lysosomes

## Abstract

Membrane contact sites (MCSs) between endosomes and the endoplasmic reticulum (ER) are thought to act as specialized trigger zones for Ca^2+^ signaling, where local Ca^2+^ released *via* endolysosomal ion channels is amplified by ER Ca^2+^-sensitive Ca^2+^ channels into global Ca^2+^ signals. Such amplification is integral to the action of the second messenger, nicotinic acid adenine dinucleotide phosphate (NAADP). However, functional regulators of inter-organellar Ca^2+^ crosstalk between endosomes and the ER remain poorly defined. Here, we identify progesterone receptor membrane component 1 (PGRMC1), an ER transmembrane protein that undergoes a unique heme-dependent dimerization, as an interactor of the endosomal two pore channel, TPC1. NAADP-dependent Ca^2+^ signals were potentiated by PGRMC1 overexpression through enhanced functional coupling between endosomal and ER Ca^2+^ stores and inhibited upon PGRMC1 knockdown. Point mutants in PGMRC1 or pharmacological manipulations that reduced its interaction with TPC1 were without effect. PGRMC1 therefore serves as a TPC1 interactor that regulates ER-endosomal coupling with functional implications for cellular Ca^2+^ dynamics and potentially the distribution of heme.

Nicotinic acid adenine dinucleotide phosphate (NAADP) is a potent second messenger that releases Ca^2+^ from endosomes and lysosomes (1, 2, 3). NAADP-evoked Ca^2+^ release from these organelles locally controls membrane trafficking events to regulate transport throughout the endolysosomal system. This action likely underpins many of the physiological roles for NAADP in processes such as fertilization, synaptic transmission, neurite extension, angiogenesis, and cholesterol homeostasis (4, 5). Dysfunction of the NAADP signaling pathway has been shown in a growing portfolio of disease states - particularly neurodegeneration, infectious disease, and cancer such that interventions to manipulate NAADP action are receiving increased scrutiny as a potential therapeutic approach (5, 6, 7, 8).

NAADP causes Ca^2+^ release from endosomes and lysosomes by targeting ion channels known as two-pore channels (TPCs) resident within endosomes and lysosomes. TPCs are evolutionarily ancient members of the voltage-gated ion channel superfamily (9, 10), with two distinct family members—TPC1 (biased toward endosomal compartments) and TPC2 (predominantly lysosomal)—expressed in human cells. The finite size of endolysosomal Ca^2+^ stores, the relatively low permeability of TPCs for Ca^2+^, and the lack of positive feedback regulation of TPCs by Ca^2+^ would be expected to restrict NAADP-evoked Ca^2+^ signals to a local domain of action around these organelles. However, the spatial action of NAADP is extended *via* an amplification process that depends on the recruitment of Ca^2+^-sensitive Ca^2+^ release channels in the endoplasmic reticulum (ER). ER-resident Ca^2+^ channels (ryanodine receptors and IP_3_ receptors) are thought to be engaged by TPC-mediated Ca^2+^ signals at specific locales known as membrane contact sites (MCSs) where the membranes of both these cellular organelles occur in close apposition (11, 12). TPC1 is enriched at MCSs formed between late endosomes and the ER, with endosomal membranes that are in contact with the ER displaying a higher density of TPC1 than endosomal membranes that are not in contact with ER (13). Aside from their role in coupling Ca^2+^ dynamics between organelles, MCSs also play important roles in the exchange of lipids and metabolites, regulation of membrane dynamics, and various signaling processes unique to specific organelle-organelle pairings (11, 12).

Given the functional impact of Ca^2+^ amplification at these trigger zones, it is important to understand how TPC1 is enriched at MCSs to globalize NAADP action. Here we identify progesterone receptor membrane component 1 (PGRMC1), a heme-binding transmembrane protein resident in the ER, as a TPC1 interactor that regulates the amplification of NAADP-evoked Ca^2+^ signals at MCSs between endosomal and ER Ca^2+^ stores.

## Results

### PGRMC1 is a TPC1 interactor that potentiates NAADP-evoked Ca^2+^ release

**Figure 1:**
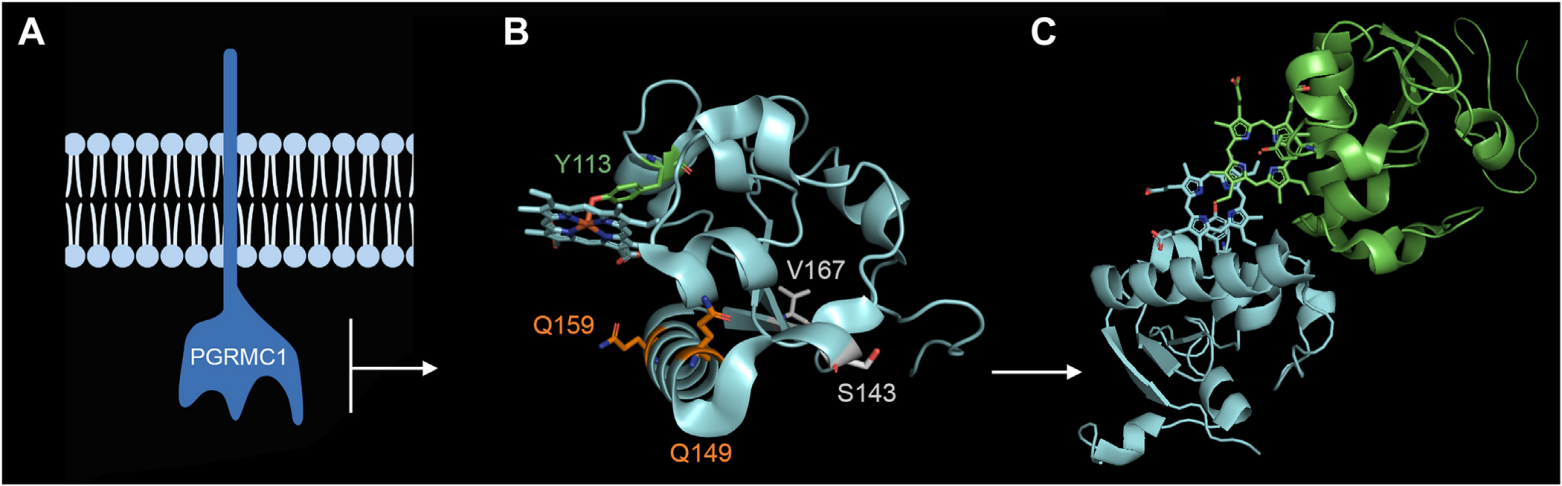
**PGRMC1 structure and interaction with TPC1.***A*, schematic illustration of PGRMC1 (195 amino acids) which is a single-pass transmembrane protein with a short NH_2_-terminal domain (amino acids 1–24), a transmembrane spanning region (residues 25–43), and a larger COOH-terminal domain (residues 44–195). The topology of PGRMC1 is debated with evidence for the COOH terminal domain residing within the cytoplasm and/or lumen of the ER. *B*, crystal structure showing amino acids 72 to 179 of PGRMC1 (*cyan*). The structure was from Protein Data Bank (PDB 4X8Y). Point mutants used in this study are highlighted to show the location with the PGRMC1 structure. These include four residues that were mutated to proline, two within the helical region (Q149 and Q159, *orange*) and two outside this region (S143 and V167, *white*). The heme coordinating residue Y113 is also highlighted (*green*). *C*, dimerization of PGRMC1 monomers *via* heme stacking as shown in (22).

PGRMC1 is a single-pass transmembrane protein (195 amino acids, Fig. 1*A*), found in the endoplasmic reticulum that has been implicated in various cellular functions (14, 15, 16, 17, 18, 19), including modulation of cellular Ca^2+^ homeostasis (20, 21). PGRMC1 acts as a heme-binding chaperone, able to dimerize through a unique heme-heme stacking mechanism. Crystallization of the COOH-terminal region of PGRMC1 (amino acids 72–179, Fig. 1*B*)) resolved the structural basis for this heme-dependent dimerization (22). Tyrosine 113 (Y113) acts as an axial ligand coordinating the heme iron within the PGRMC1 monomer. One heme-bound PGRMC1 monomer then associates with a second heme-bound PGRMC1 monomer *via* hydrophobic interactions between the two heme groups (Fig. 1*C*).

**Figure 2:**
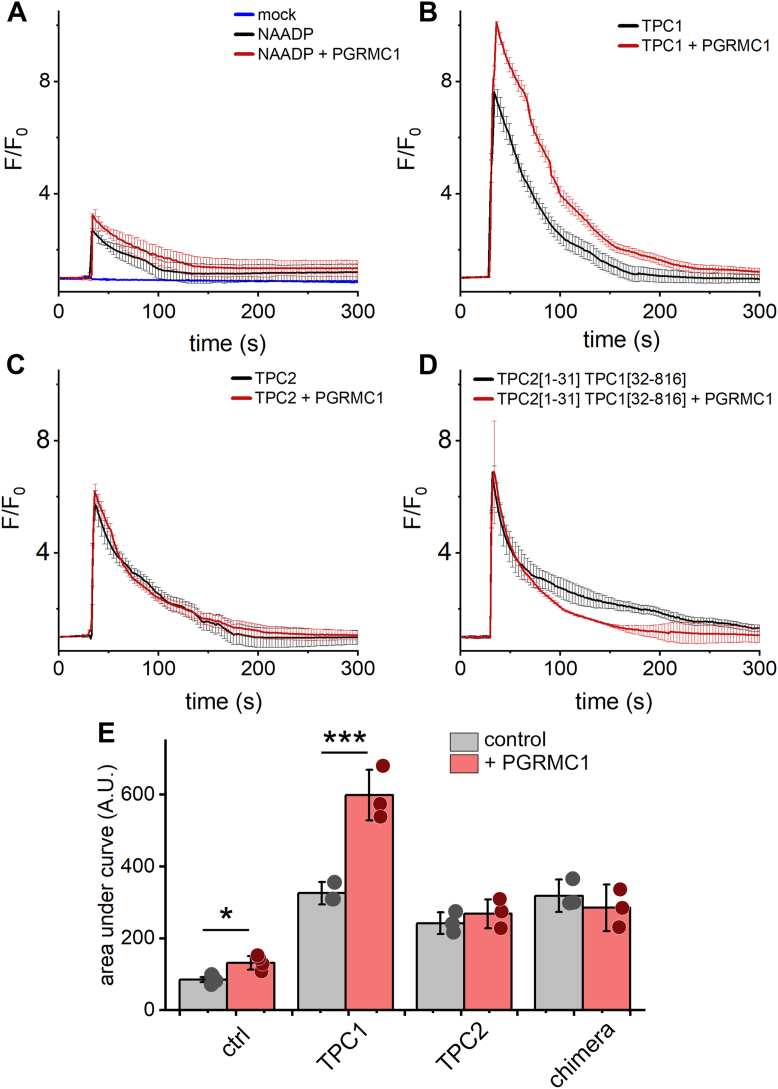
**Expression of PGRMC1 potentiates NAADP-evoked Ca**^**2+**^**release *via* TPC1.***A*, fluorescence traces from single U2OS cells microinjected with buffer (“mock injection,” *blue*) or NAADP (100 nM pipette concentration) in the absence (*black*) or presence (*red*) of RFP-PGRMC1. *B*–*D*, similar microinjection experiments in single U2OS cells expressing (*B*) TPC1, (*C*) TPC2 or (*D*) TPC2[1-31]-TPC1[32-816] in the absence (*black*) or presence (*red*) of RFP-PGRMC1. Traces report the fluorescence of GCaMP6M over time-averaged (mean ± sd) from n ≥ 3 injected cells, with only every third data point shown for clarity. *E*, area under the curve from NAADP-evoked Ca^2+^ mobilization responses, values represent mean area ± sd from independent fluorescence traces recorded from n ≥ 3 injected cells. Statistical significance was determined using paired two-tailed Student’s *t* test, *p*-values, ∗*p* < 0.05, ∗∗∗*p* < 0.001.

Our prior proteomic analysis of the TPC interactome identified PGRMC1 as a candidate TPC1 interacting protein (Fig. S1, (23)). To assess any functional consequences of this interaction, Ca^2+^ imaging experiments were performed to assess the impact of PGRMC1 on NAADP-evoked Ca^2+^ release. This was examined by microinjecting NAADP into individual U2OS cells, an osteosarcoma-derived cell line routinely used for studying NAADP-evoked Ca^2+^ release (24, 25). In U2OS cells overexpressing PGRMC1 (RFP-PGRMC1), endogenous NAADP-evoked Ca^2+^ signals appeared larger compared with responses to NAADP in control (untransfected) cells (Fig. 2*A*). Owing to the small amplitude of these endogenous NAADP-evoked Ca^2+^ signals, responses were also examined in cells overexpressing TPC1 (biased toward endosomes) or TPC2 (biased toward lysosomes), a known manipulation that enhances NAADP-evoked Ca^2+^ signals (26). In cells overexpressing TPC1, NAADP-evoked Ca^2+^ signals became more pronounced than in control cells (Fig. 2*B*). Overexpression of RFP-PGRMC1 in this background further potentiated the NAADP-evoked Ca^2+^ mobilization (Fig. 2*B*). Potentiation of NAADP responses by PGRMC1 was not observed in cells overexpressing TPC2 (Fig. 2*C*). These data are consistent with the observed association of PGRMC1 with TPC1, but not TPC2 (Fig. S1). An endosomal localization of TPC1 was necessary to support the observed potentiation of NAADP-evoked Ca^2+^ signals by PGRMC1. In cells where TPC1 was rerouted to a lysosomal location by use of a chimeric construct (TPC2[1-31]:TPC1[32-816)), which contains the TPC1 backbone fused to the NH_2_-sequence of TPC2 that harbors lysosomal-targeting determinants, overexpression of PGRMC1 did not potentiate NAADP-evoked Ca^2+^ release (Fig. 2*D*). Quantitative analysis of these experiments is provided in Figure 2*E*. These results demonstrate that PGRMC1 acts to potentiate NAADP-evoked Ca^2+^ signals *via* TPC1.

### Mutational ablation of the interaction between PGRMC1 and TPC1 blocks potentiation of NAADP-evoked Ca^2+^ release

Mutational analysis was then performed with a goal of identifying mutants that disrupt the PGRMC1-TPC1 interaction. Residues 147 to 163 of PGRMC1 form an α-helical domain (Fig. 1*B*) that potentially serves as a protein interaction interface. Five PGRMC1 point mutants were generated (Fig. 1*B*): the PGRMC1 dimerization breaking mutant Y113F (PGRMC1[Y113F] (22)), and four different proline mutants, two within the α-helical domain (PGRMC1[Q149P] and PGRMC1[Q159P]) and two outside this region (PGRMC1[S143P] and PGRMC1[V167P]).

**Figure 3:**
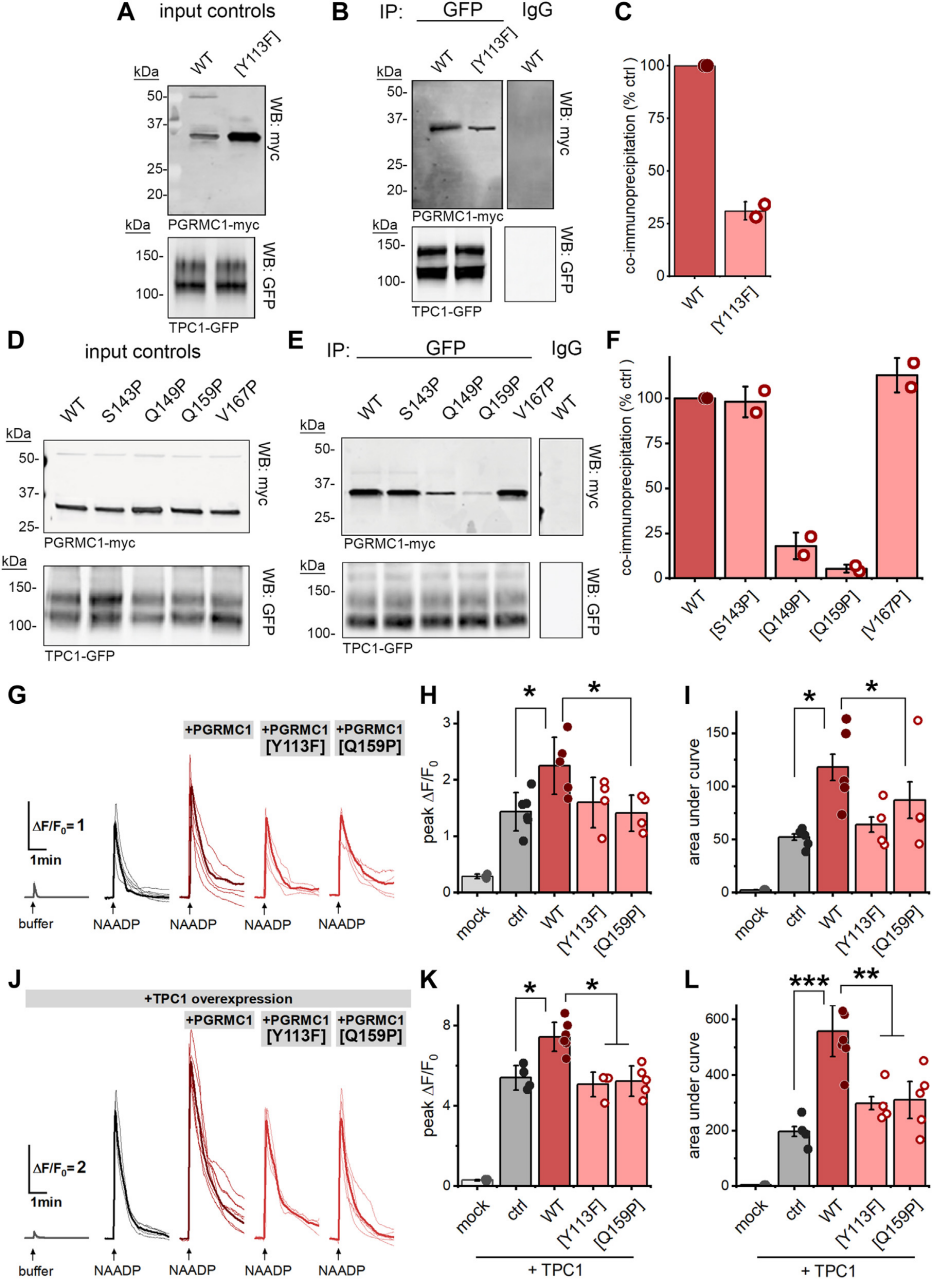
**Molecular disruption of the PGRMC1-TPC1 interaction impairs PGRMC1 potentiation of NAADP-evoked Ca**^**2+**^**release.***A*–*C*, heme binding is necessary for interaction with TPC1. *A*, Myc-tagged WT PGRMC1 and PGRMC1[Y113F] constructs were co-expressed in U2OS cells with GFP-tagged TPC1, with detection by Western blot. *B*, immunodetection of myc-tagged PGRMC1 after TPC1-GFP immunoprecipitation. *C*, densitometry quantification of immunodetection of myc-tagged PGRMC1 in TPC1-GFP immunoprecipitates. *D*–*F*, interaction of PGRMC1 with TPC1 is mediated by the COOH-terminal coiled-coil domain of PGRMC1. *D*, PGRMC1 mutants harboring point-mutations in predicted carboxyl-terminus helices co-expressed with TPC1-GFP in U2OS cells, detected by Western blot. *E*, immunodetection of myc-tagged PGRMC1 mutants after TPC1-GFP immunoprecipitation. *F*, densitometry quantification of immunodetected myc-tagged PGRMC1 mutants in TPC1-GFP immunoprecipitates, values represent mean ± sd from n = 2 independent experiments. *G*, traces of Ca^2+^ flux in U2OS cells expressing the indicated PGRMC1 constructs microinjected with NAADP (100 nM pipette concentration) as detected by GCaMP6M fluorescence changes. Individual single-cell traces from n ≥ 3 injections are shown, with averaged responses bolded. *H*, peak F/F_0_ values (mean ± sd) from cumulative dataset shown in (*G*). *I*, area under the curve (mean ± sd) of traces shown in (*G*). *J*, averaged traces of Ca^2+^ flux in U2OS cells expressing TPC1-RFP and the indicated PGRMC1 constructs microinjected with NAADP (100 nM pipette concentration) as detected by GCaMP6M fluorescence changes. Individual single-cell traces from n ≥ 3 injections are shown, with averaged responses bolded. *K*, peak F/F_0_ values (mean ± sd) from cumulative dataset shown in (*J*). *L*, area under the curve (mean ± sd) of traces shown in (*J*). Statistical significance was determined using paired two-tailed Student’s *t* test, *p*-values, ∗*p* < 0.05, ∗∗∗*p* < 0.001.

The PGRMC1[Y113F] mutant, which prevents heme binding to PGRMC1 and thereby PGRMC1 dimerization, caused an increase in monomeric PGRMC1 (Fig. 3*A*). Apo-PGRMC1 displayed decreased interactivity with TPC1 (Fig. 3*B*). Quantitative analysis of these experiments are shown in Figure 3*C*. Two of the proline point mutants (PGRMC1[Q149P], PGRMC1[Q159P]), also displayed decreased interaction with TPC1. PGRMC1[Q159P] conferred the strongest inhibition (Fig. 3, *D* and *E*). Interestingly both these mutants lie within the helical domain of PGRMC1 (Fig. 1*B*). In contrast, the proline point mutants designed outside the PGRMC1 helical domain failed to disrupt the interactivity between PGRMC1 and TPC1 (Fig. 3, *D* and *E*). Quantitative analysis of these immunoprecipitation assays is provided in Figure 3*F*. These data demonstrate that PGRMC1 associates with TPC1, and this interaction is selectively attenuated in cells when the heme binding and dimerization of PGRMC1 is prevented (PGRMC1[Y113F]), or when a helix-breaking mutant is introduced within the PGRMC1 helical domain (PGRMC1[Q159P]).

Given the strong impact of each of these classes of mutants on TPC1 interactivity, the functional effect of PGRMC1[Y113F] and PGRMC1[Q159P] was probed using Ca^2+^ imaging assays. Overexpression of PGRMC1[Y113F] or PGRMC1[Q159P] in U2OS cells failed to potentiate endogenous NAADP-evoked Ca^2+^ signals (Fig. 3, *G*–*I*). Similarly, in U2OS cells overexpressing TPC1, the NAADP-evoked Ca^2+^ mobilization response was not potentiated by expression of PGRMC1[Y113F] or PGRMC1[Q159P] in contrast to the potentiation seen with wild type PGRMC1 (Fig. 3, *J*–*L*). The PGRMC1 [S143P] mutation, which failed to disrupt TPC1 interactivity, retained potentiation of NAADP-evoked signaling, similar to wild-type PGRMC1 (Fig. S2). Additionally, knockdown of endogenous PGRMC1 using gene-specific siRNA resulted in an attenuation of NAADP-evoked Ca^2+^ signals (Figs. S2 and S3). Finally, a U2OS TPC1-knockout cell line was used to assess the effects of PGRMC1 overexpression in the absence of TPC1. In the TPC1 KO cell line, injection of NAADP was without effect, as expected. TPC1 KO cells overexpressing PGRMC1 remained unresponsive to NAADP, suggesting that PGRMC1 cannot independently mobilize Ca^2+^ in response to NAADP (Fig. S2). Therefore, potentiation of NAADP action by PGRMC1 depends on the ability of PGRMC1 to interact with TPC1.

### Pharmacological inhibition of the interaction between PGRMC1 and TPC1 blocks potentiation of NAADP-evoked Ca^2+^ release

As an alternative means of study the functional impact of PGRMC1 interactivity with TPC1, we employed a pharmacological approach. Two PGRMC1 inhibitors were used: AG-205 and CORM3. AG-205, which competes with heme to bind the cytochrome b5 domain is widely used as an inhibitor of PGRMC1, although we note this drug has a broader polypharmacology (27, 28, 29, 30). CORM3 (tricarbonylchloro(glycinato)ruthenium(II)) is a carbon monoxide–releasing agent, which blocks PGRMC1 dimerization by binding to the open coordination site in PGRMC1-bound heme (16).

**Figure 4:**
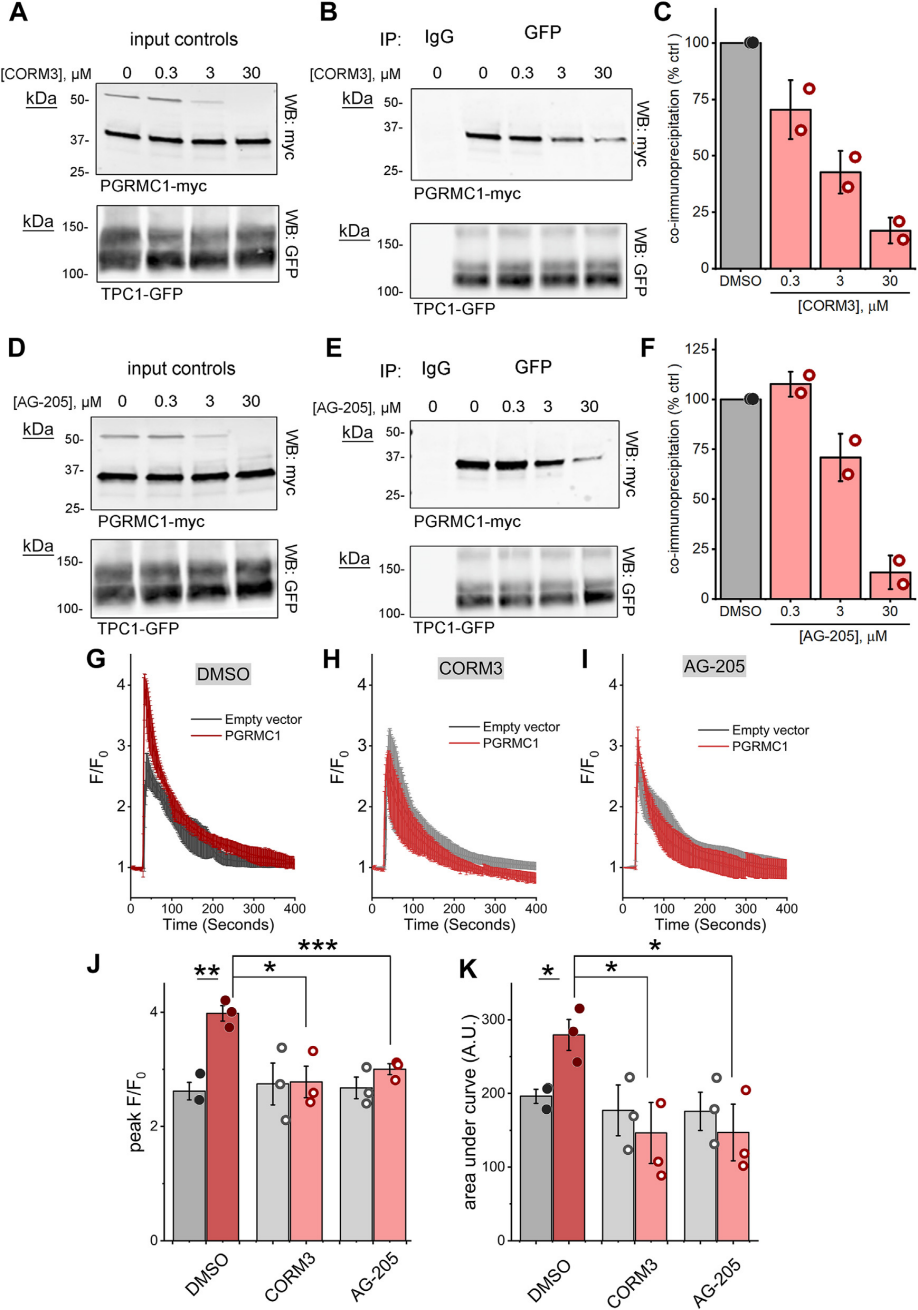
**Pharmacological disruption of the PGRMC1-TPC1 interaction blocks PGRMC1 potentiation of NAADP-evoked Ca**^**2+**^**release.***A* and *D*, immunodetection of PGRMC-myc and TPC1-GFP in lysates from U2OS cells pretreated for 1 h with the indicated concentration of CORM3 (*A*) or AG-205 (*D*). *B* and *E*, immunodetection of PGRMC1-myc after immunoprecipitation of TPC1-GFP from lysates shown in (*A* and *D*). *C* and *F*, densitometric quantification of myc-tagged PGRMC1 from TPC1-GFP immunoprecipitates, values represent mean ± sd from n = 2 experiments. *G*–*I*, averaged fluorescence traces (mean ± sd from n ≥ 3 injections) showing NAADP responses in U2OS cells transfected with empty vector (*black*) or PGRMC1 (*red*, pretreated with (*G*) DMSO (0.1%), (*H*) CORM3 (30 μM), or (*I*) AG-205 (30 μM) for 1 h, detected by following GCaMP6M fluorescence. *J*, peak F/F_0_ values (mean ± sd) of independent NAADP responses summarized in (*G*–*I*). *K*, area under the curves (mean ± sd) of independent traces of NAADP-responses summarized in (*G*–*I*). Statistical significance was determined using paired two-tailed Student’s *t* test, *p*-values, ∗*p* < 0.05, ∗∗*p* < 0.01, ∗∗∗*p* < 0.001.

Immunoprecipitation analyses were performed following the treatment of cells with increasing concentrations of either agent. Increasing concentrations of CORM3 caused a concentration-dependent inhibition of the PGRMC1 interaction with TPC1 as evidenced by the decrease in the ∼37 kDa PGRMC1 band in the TPC1 immunoprecipitates (Fig. 4, *A*–*C*). A similar concentration inhibition was observed in cells exposed to AG-205 (Fig. 4, *D*–*F*). Peak impairment of PGRMC1-TPC1 interactivity was observed following exposure to either drug (30 μM) for 1 h. These conditions were subsequently employed in functional assays of NAADP action. Under the same conditions, exposure to either drug inhibited the PGRMC1-dependent potentiation of endogenous NAADP-evoked Ca^2+^ signals (Fig. 4, *G*–*I*). After drug treatment, no potentiation of the peak amplitude (Fig. 4*J*) or extent of NAADP-evoked Ca^2+^ release was seen (Fig. 4*K*). These results, consistent with the PGRMC1 mutational analysis described earlier (Fig. 3), again demonstrate that inhibition of the interaction of PGRMC1 with TPC1 prevents the potentiation of NAADP-evoked Ca^2+^ signals.

### PGRMC1 localizes to the endoplasmic reticulum

**Figure 5:**
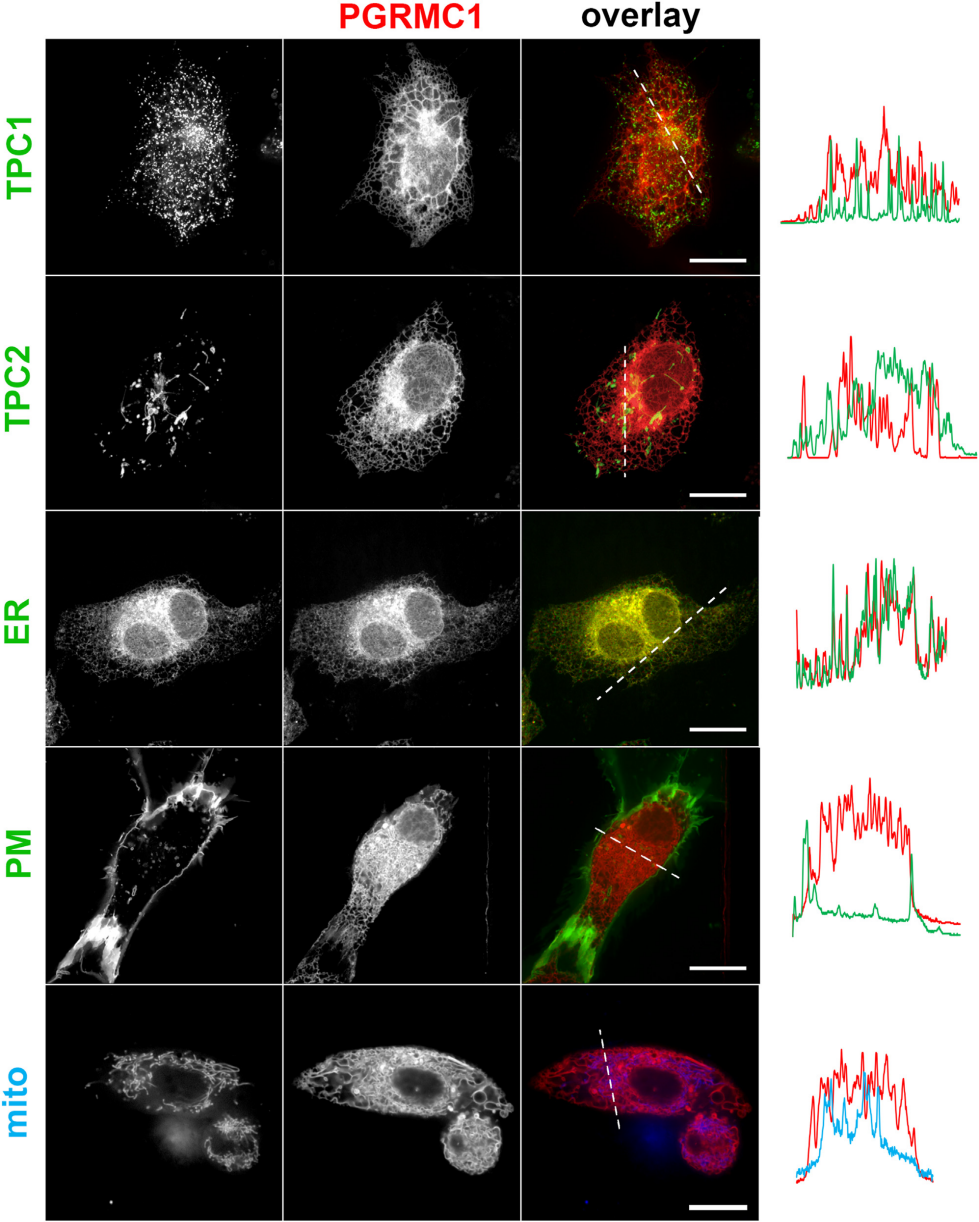
**PGRMC1 localizes to the endoplasmic reticulum in U2OS cells.***A*, U2OS cells imaged using various live cell markers (*left*) and co-transfected with PGRMC1-RFP (*center*). The fluorescence overlay of PGRMC1-RFP and the organelle marker channel is shown on the *right*. Markers were TPC1-GFP, TPC2-GFP, an endoplasmic reticulum marker (CYP2C9[1-27]-GCaMP-6M), a plasma membrane marker (GAP43[1-20]-GFP) and a mitochondrial marker (COX8A[1-29]-CFP), Scale bar, 10 μm. *Right*, *dashed line* represents a line scan of fluorescence intensity from the *green*, *blue* or *red* fluorescence channels along the indicated axis. Gamma values were uniformly adjusted for clarity.

While PGRMC1 can display a broad distribution in cells (17, 18, 19), it is most consistently considered as an endoplasmic reticulum (ER) resident protein. PGRMC1 localization in U2OS cells was therefore examined by multicolor imaging in live cells. Co-expression of PGRMC1-RFP with TPC1-GFP, TPC2-GFP, or an ER marker (KDEL-GFP) revealed a clear co-localization between PGRMC1-RFP and KDEL-GFP (Fig. 5). In contrast, no co-localization was seen between mitochondrial and plasma membrane markers, COX8A[1-29]-CFP and GAP43[1-20]-GFP, respectively (Fig. 5). Therefore, PGRMC1 behaves as an ER-resident protein in these experiments.

### PGRMC1 potentiation of NAADP action results from enhanced mobilization of ER Ca^2+^

**Figure 6:**
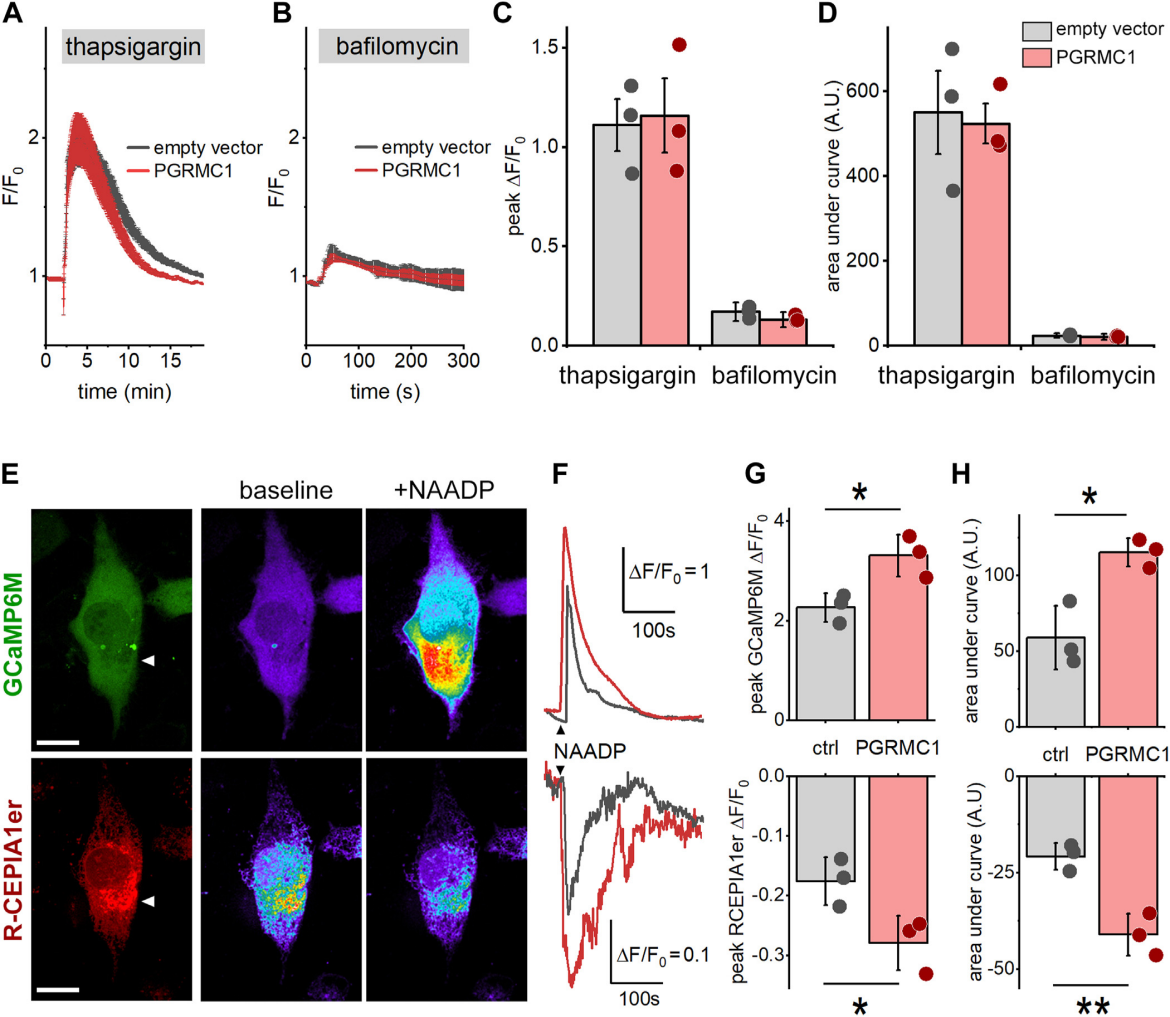
**Expression of PGRMC1 increases NAADP-evoked Ca**^**2+**^**release from the endoplasmic reticulum.***A* and *B*, averaged fluorescence traces as detected by GCaMP6M fluorescence changes reporting NAADP-evoked Ca^2+^ release in U2OS cells transfected with empty vector or in cells expressing PGRMC1. Microinjections were performed with NAADP (100 nM pipette concentration) in the presence of (*A*) thapsigargin (1 μM) or (*B*) bafilomycin A1 (100 nM). *C*, quantification of peak ΔF/F_0_ values from (*A* and *B*). *D*, quantification of the area under the curves from (*A* and *B*). *E*, *left*, Images of U2OS cell co-expressing GCaMP6M (*top*) and RCEPIA1er (*bottom*). *Middle*, pseudocolored images of GCaMP6M and RCEPIA1er fluorescence intensity before (middle image) and after (*right* image) microinjection of NAADP. Scale bar,10 μm. *F*, representative traces of GCaMP6M fluorescence (*top*) and RCEPIA1er fluorescence (*bottom*) in response to microinjection of NAADP (100 nM pipette concentration) in U2OS cells transfected with empty vector (*grey*), or from cells expressing PGRMC1 (*red*). *G*, quantification of ΔF/F_0_ values from the cumulative dataset for GCaMP6M (*top*) and RCEPIA1er fluorescence (*bottom*). *H*, quantification of area under the curves from the cumulative dataset for GCaMP6M (*top*) and RCEPIA1er fluorescence (*bottom*). Statistical significance was determined using paired two-tailed Student’s *t* test, *p*-values, ∗*p* < 0.05, ∗∗*p* < 0.01.

Does PGRMC1 enhance the size of the Ca^2+^ stores mobilized by NAADP? As NAADP-evoked Ca^2+^ release from acidic Ca^2+^ stores is amplified through the recruitment of Ca^2+^-sensitive channels in the ER, the size of both the acidic Ca^2+^ stores and ER Ca^2+^ stores were resolved in control cells and cells expressing PGRMC1. This was performed by treatment of U2OS cells with thapsigargin (Fig. 6*A*) or bafilomycin A1 (Fig. 6*B*) respectively. These experiments revealed no difference in the magnitude of either Ca^2+^ store between control cells and cells expressing PGRMC1 (Fig. 6, *C* and *D*). NAADP-evoked changes in cytosolic and ER Ca^2+^ were then examined using dual emission imaging to simultaneously resolve both the cytosolic Ca^2+^ signal and luminal ER Ca^2+^ dynamics. In U2OS cells expressing two genetically encoded Ca^2+^ indicators (Fig. 6*E*), the kinetics of the endogenous NAADP-evoked cytosolic Ca^2+^ signal (resolved using GCaMP6M) closely paralleled the kinetics of luminal ER Ca^2+^ depletion (resolved using RCEPIA1er). Following PGRMC1 expression, both signals increased in magnitude, with PGRMC1 expression associated with a larger, more protracted cytoplasmic Ca^2+^ signal in response to microinjection of NAADP (Fig. 6*F*). This potentiation was also observed at the level of the ER, with the larger cytoplasmic Ca^2+^ signal correlating with a larger depletion of ER Ca^2+^ (Fig. 6*E*). These effects were quantified in Figure 6, *G* and *H*. Therefore, the potentiating action of PGRMC1 on NAADP-evoked Ca^2+^ signals is manifested through enhanced mobilization of ER Ca^2+^.

**Figure 7:**
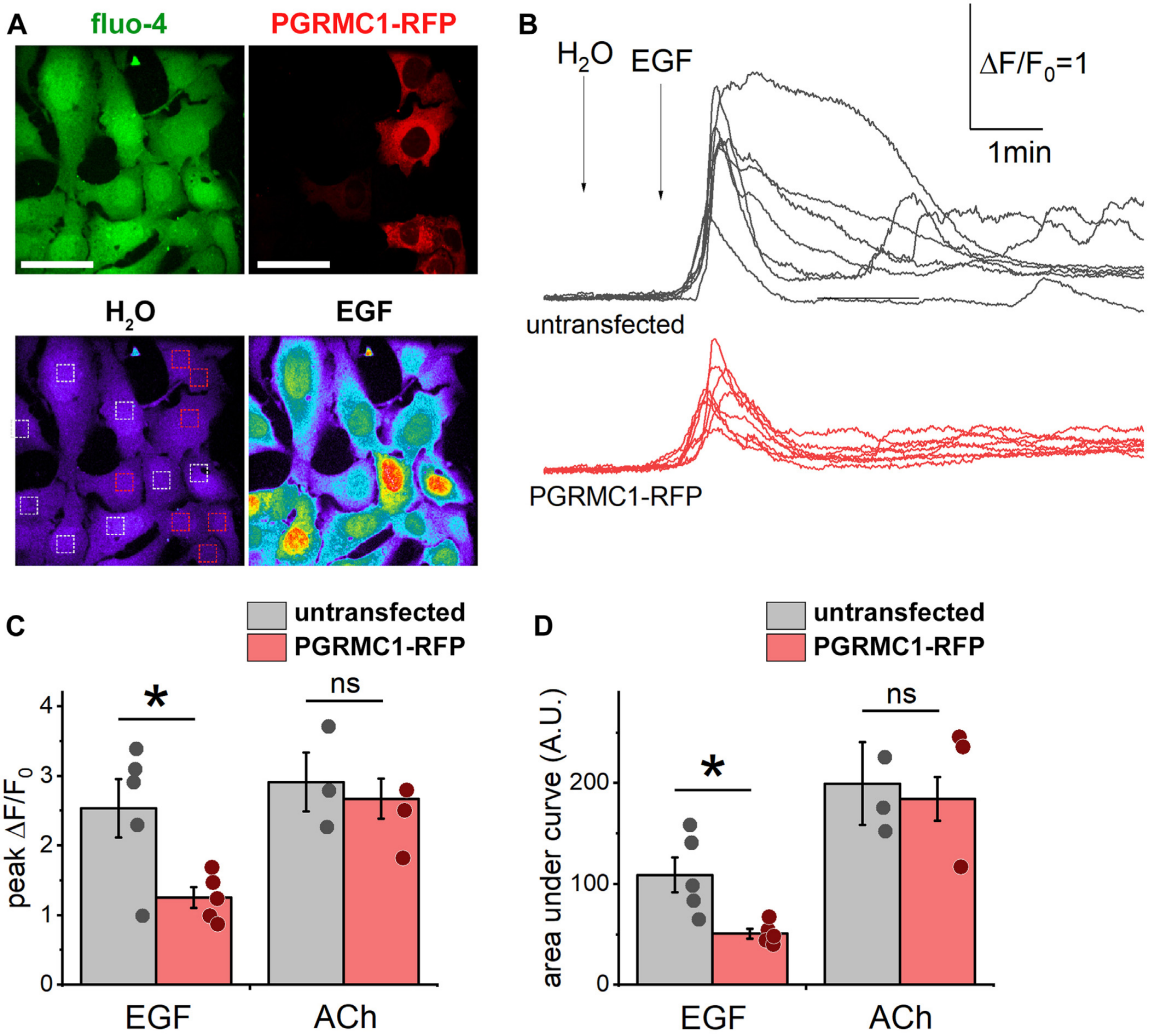
**Expression of PGRMC1 inhibits EGF-evoked Ca**^**2+**^**release.***A*, U2OS cells were transfected with vector encoding PGRMC1-RFP (*top right*) and loaded with fluo-4 dye (*top left*). Pseudocolored images of fluo-4 fluorescence intensity after addition of buffer (*bottom*, *left*) or EGF (32 PM, *bottom*, *right*). Kinetics of Ca^2+^ signals (fluo-4 fluorescence) from U2OS cells expressing PGRMC1-RFP (*red boxes*) and untransfected cells (*white boxes*) were compared. Scale bar, 20 μm. *B*, single-cell fluorescence traces from EGF responses in U2OS cells in the absence (*black*, *top*) or presence of PGRMC1-RFP (*red*, *bottom*). *C* and *D*, quantification of (*C*) peak F/F_0_ or (*D*) area under the curve (AUC) in response to EGF (32 PM) or acetylcholine (ACh, 100 μM) in control U2OS cells (*black*) or cells overexpressing PGRMC1 (*red*). Data are shown as average±sem. Statistical significance was determined using paired two-tailed Student’s *t* test, *p*-values, ∗*p* < 0.05; ns, not significant.

A parsimonious explanation for this effect is that PGRMC1-TPC1 interaction between PGRMC1 (localized in the ER) and TPC1 (localized in endosomes) enhances the functional coupling between NAADP-activated TPC1 channels in endosomes and Ca^2+^-activated Ca^2+^ release channels within the ER. Specifically, PGRMC1 may increase the number (or longevity) of membrane contact sites (MCSs) between TPC1-positive endosomes and the ER. One method for assessing the functional coupling between these organelles is through the measurement of EGF-evoked Ca^2+^ signals (13, 31). The kinetics of EGFR-evoked Ca^2+^ signals are regulated by the phosphorylation status of the EGFR. The tyrosine phosphatase, PTP1B, which dephosphorylates the EGFR is localized on the ER, with dephosphorylation of the EGFR dampening Ca^2+^ release activity. Therefore, the structural intimacy between endosomes and the ER dictates EGFR phosphorylation status and thereby the magnitude of EGF-evoked Ca^2+^ signals. Decreased EGF-mediated Ca^2+^ signals reflect an enhanced proximity between these organelles (31). EGF-evoked Ca^2+^ responses were therefore compared between control U2OS cells and U2OS cells expressing PGRMC1-RFP (Fig. 7*A*). Responses to EGF in control U2OS cells were robust and were dampened in those cells expressing PGRMC1 (single cells identified by RFP fluorescence, Fig. 7*B*). Both the peak magnitude and size (AUC) of EGF-evoked Ca^2+^ signals were depressed in PGRMC1-expressing cells (Fig. 7, *C* and *D*). However, no changes in Ca^2+^ signals evoked by acetylcholine were noted between the same cells (Fig. 7, *C* and *D*). Therefore, by using EGF activity as a reporter for endosome-ER connectivity, these data demonstrate that the interaction between PGRMC1 and TPC1 enhanced functional Ca^2+^ crosstalk between TPC1-positive endosomes and the ER.

## Discussion

Here we demonstrate that PGRMC1 interacts with TPC1 to regulate functional crosstalk between ER and endosomal Ca^2+^ stores. Overexpression of PGRMC1 enhanced NAADP-evoked Ca^2+^ signals (Fig. 2) by causing a greater mobilization of ER Ca^2+^ (Fig. 6). Loss of PGRMC1-TPC1 interactivity, effected using mutational or pharmacological strategies (Figs. 3 and 4), prevented this potentiation. Knockdown of endogenous PGRMC1 also inhibited NAADP-evoked Ca^2+^ signals (Fig. S2). This role for PGRMC1 in regulating the gain of NAADP-evoked Ca^2+^ signaling is novel, although PGRMC1 has been implicated in many facets of cellular physiology that show overlap with established roles for NAADP (14, 15, 16, 17, 18, 19). Such roles include endocytic trafficking (32, 33, 34), insulin homeostasis (35, 36), lipid homeostasis (34, 37), as well as axonal guidance and synaptogenesis (14). PGRMC1 is also implicated in various pathophysiological outcomes such as non-alcoholic fatty liver disease (38), diabetes (39) and cancer (22, 29, 40, 41), which parallel the emerging role of the TPC complex in many of the same disease states (5, 7, 8). We note PGRMC1 is highly expressed in several cancer types, serving to accelerate tumor progression and being associated with poor clinical prognosis. Potentially, the coupling of NAADP–evoked Ca^2+^ release to ER Ca^2+^ signals is also enhanced by the elevated levels of PGRMC1 in these tumors, and this may contribute to the pathological events driving carcinogenesis (42).

Our data evidencing a functional role for PGRMC1 at endosome-ER membrane contact sites is further supported by the recent identification of PGRMC1 as a reticulon-3 binding (RTN3) partner (43). RTN3 regulates endosomal maturation at sites of endosomal-ER contact (44). PGRMC1 has also been localized at other types of MCSs formed between the ER and other organelles. These include ER-plasma membrane junctions (21) and ER-mitochondria contacts (45).

A key facet of PGRMC1 biology is the ability of PGRMC1 to bind heme (K_d_ of 50 nM, (22)) and then dimerize *via* a unique heme-heme stacking interaction, mediated *via* hydrophobic interactions between the heme moieties of two heme-bound PGRMC1 monomers. Therefore, while we have shown that PGRMC1 regulates Ca^2+^ signaling between endosomes and the ER, the localization of PGRMC1 at ER-endosomal junctions may also have significance for the cellular transport of heme. The cytoplasmic concentrations of free heme, just like free Ca^2+^, must be tightly regulated to avoid cellular toxicity. After synthesis inside mitochondria, heme must be distributed throughout the cell to fulfill various biological roles (46, 47). It has been proposed that the ER may act as an efficient conduit for heme distribution, a concept supported experimentally through the use of genetically encoded heme biosensors (48, 49). PGRMC1 has previously been implicated as a chaperone for mediating heme efflux from mitochondria at contact sites between ER and mitochondria (46, 48, 50). PGRMC2 also plays a role in heme trafficking between mitochondria and the nucleus (51). Our data could implicate a role for PGRMC1 at endosomal-ER contacts to mediate the transfer of heme between the ER and the endosomal system. The hydrophobic nature of heme makes shuttling between heme-binding proteins clustered at MCSs a feasible solution for heme transfer that avoids elevation of the cytosolic concentration of labile heme. Mutation of the tyrosine residue Y113 (Y113F) decreased PGRMC1 interactivity with TPC1 (Fig. 3*B*). This residue, as a tyrosinate, acts as the 5-coordinating ligand for the heme iron atom (16) such that the Y113F mutant prevents heme binding and PGRMC1 dimerization. Heme association with PGRMC1 may therefore increase affinity for TPC1, coupling changes in local ER heme concentration to MCS formation.

The interaction between PGRMC1 and TPC1 establishes a regulable nexus supporting crosstalk between discrete organelles. Exploration of cues that impact PGRMC1 properties in the context of MCS function has merit for future study. These include physiological levels of carbon monoxide, which dissociate the PGRMC1 dimer, cancer-associated mutants in PGRMC1, as well as changes in PGRMC1 phosphorylation or protein-protein interactions.

One particular PGRMC1 interaction that is especially intriguing is with STIM1, the ER Ca^2+^ sensor that activates store-operated Ca^2+^ entry following ER Ca^2+^ store depletion (21). This forms part of a growing body of literature concerning PGRMC1 and Ca^2+^ entry (20, 21). PGRMC1, and PGRMC1[Y113F], translocate on Ca^2+^ store depletion through association with the coiled-coil domain of STIM1 to interact with Orai1 channels at ER-plasma membrane junctions (21). That an endosome-ER MCS component (regulating Ca^2+^ store depletion) transitions to a cell surface-ER junction (regulating Ca^2+^ store refilling) underscores the dynamic nature of MCS components and differential functional roles that are keyed to their specific spatial localization.

In conclusion, these data demonstrate a molecular interaction between PGRMC1 and TPC1 that regulates the amplitude and duration of NAADP-evoked Ca^2+^ signals. This role establishes PGRMC1 function at an MCS interface, between endosomes and the ER, where PGRMC1 acts as a regulatory adaptor impacting Ca^2+^ dynamics and potentially heme transport.

## Experimental procedures

### Drugs and molecular reagents

CORM-3, AG-205, bafilomycin A1, thapsigargin, and acetylcholine chloride were from Sigma Aldrich. The recombinant human epidermal growth factor was from BioVision (Waltham, MA). NAADP was synthesized in-house by incubating nicotinamide adenine dinucleotide phosphate (NADP, Sigma Aldrich) with nicotinic acid (Sigma Aldrich) in the presence of *Aplysia* ADP-ribosyl cyclase and purified by high-performance liquid chromatography, as previously described (52). The following plasmids were sourced from Addgene (Watertown, MA): pcDNA3-Clover (plasmid# 40259), pGP-CMV-GCaMP6M (plasmid# 40754), and pCMV-R-CEPIA1er (plasmid# 58216). pDONR221 vector containing cDNA encoding PGRMC1 (accession NM_006667.3) was sourced from DNASU (Clone ID FLH179805.01L). PGRMC1 cDNA was subcloned into pCS2+Myc and pCS2+mRFP using ClaI and EcoRI restriction sites. PGRMC1 point mutants were made by PCR-based mutagenesis. pCS2+ vectors containing TPC sequences with either 5x Myc, EGFP or mRFP tags have been previously described (23), and were used as templates to subclone TPC1 and TPC2 cDNA into pcDNA3.1(+)-Clover at the EcoRI restriction site. PGRMC1-specific siRNA (catalog #4390824, siRNA ID s21310, 5′ – CAGUUCACUUUCAAGUAUCtt- 3′) was sourced from Thermo Fisher (Waltham MA). Endoplasmic reticulum marker (CALR[1-17]-GFP-KDEL) was sourced from Invitrogen, plasma membrane marker (GAP43[1-20]-GFP) and mitochondrial marker (COX8A[1-29]-CFP) were originally sourced from Clontech. Antibodies were sourced and utilized as follows: rabbit anti-PGRMC1 (0.2 μg/ml; Santa Cruz Biotechnology, sc-393015), mouse anti-GFP antibody (0.2 μg/ml; Santa Cruz Biotechnology, sc-9996), rabbit anti-myc antibody (0.2 μg/ml; Thermo Fisher Scientific; PA5-85185). Antibody specificity was validated by probing membranes in the absence of targeted overexpressed proteins, or after knockdown of targeted endogenous protein. IRDye near-infrared secondary antibodies (0.1 μg/ml; Li-COR Biosciences) were used for fluorescent immunodetection.

### Cell culture and transfection

Wild-type parental U2OS cells (RRID:CVCL_0042) were sourced from ATCC. U2OS cells were maintained in Dulbecco’s Modified Essential Medium (DMEM, supplemented with 10% FBS and 100 U/ml penicillin and streptomycin) and cultured in a humidified incubator (5% CO_2_ at 37 °C). U2OS TPC1 knockout cells were generated in collaboration with Synthego (Redwood City, CA), using CRISPR-Cas9 with three independent guide RNAs targeting exon 4 (sgRNA1, CGCCAGCGCCUUGGCAUCCU; sgRNA2, GGGCCGUGGCCAGCUCCAUC; sgRNA3, CCGGAGUGCGGGGACGGCGG). Deletion of exon 4 was validated by Sanger sequencing of isolated genomic DNA. For overexpression experiments, U2OS cells were transfected with constructs using Lipofectamine 3000 (Thermo Fisher Scientific). Briefly, 3 × 10^6^ cells were seeded in a 75 cm^2^ flask in the absence of antibiotics. The following day, transfection complexes were produced by combining plasmid DNA (15 μg), P3000 reagent (30 μl) and Lipofectamine 3000 reagent (20 μl), consistent with the vendor protocol. The DNA-lipid complexes were then added to cells and incubated overnight. Cells to be used the following day for imaging experiments were trypsinized and reseeded into MatTek dishes (Ashland, MA). Cells being used for biochemical experiments were harvested 48 h post transfection. All cells are free of *mycoplasma* contamination and are routinely tested using standard PCR-based methods.

### Immunoblotting and immunoprecipitations

To prepare cell lysates, U2OS cells were washed in PBS, harvested by mechanical scraping, and then centrifuged at 800×RCF. Cell pellets were suspended in a lysis buffer consisting of 110 mM KCl, 10 mM NaCl, 10 μM CaCl_2_, 20 mM HEPES (pH 7.2), 1% Triton X-100, complete EDTA-free protease inhibitor cocktail (Roche), and Halt phosphatase inhibitor cocktail (Pierce). Samples were lysed on a nutating mixer for 30 min at 4 °C prior to centrifugation at 16,000×RCF for 10 min at 4 °C. Soluble supernatants were collected, and protein concentrations were determined by Bradford assay (Thermo Fisher Scientific). For immunodetection of proteins, a sample (20 μg) of solubilized lysate was separated by SDS-PAGE and transferred to nitrocellulose membranes using standard methods. Nitrocellulose membranes were blocked in 5% skim milk in Tris-buffered saline supplemented with 0.1% Tween 20 (TBST) for 1 h at room temperature. Membranes were then incubated with primary antibodies (mouse anti-GFP antibody, 0.2 μg/ml; rabbit anti-PGRMC1, 0.2 μg/ml) overnight at 4 °C. The following day, membranes were washed three times in TBST, before incubating membranes with IRDye secondary antibodies (1:7000 dilution in 5% skim milk TBST) for 1 h at room temperature. Membranes were processed using a Li-COR Odyssey Imaging system. For co-immunoprecipitation experiments with TPCs, 1 mg of solubilized protein from U2OS cell lines overexpressing TPC1-GFP and PGRMC1-myc was incubated with either mouse IgG isotype control antibody (2 μg/ml) or anti-GFP antibody produced in rabbit (2 μg/ml; Thermo Fisher Scientific, G10362) at 4 °C for 1 h before overnight incubation with protein G agarose beads (Roche). Beads were collected after brief centrifugation and washed three times with lysis buffer. Immunoprecipitated complexes were eluted by incubating beads with 2× Laemmli sample buffer at 95 °C for 10 min and analyzed by SDS-PAGE and immunoblotting as described above.

### NAADP microinjection and Ca^2+^ imaging

Single-cell microinjections were performed as previously described (26), using an InjectMan-4 micromanipulation system and FemtoJet4i system (Eppendorf). Cells grown on glass-bottom MatTek dishes were mounted on an Olympus IX81. Dishes were perfused with Hanks Balanced Salt Solution (HBSS) without Ca^2+^ at a flow rate of 1 ml/min. Isolated cells expressing GCaMP6M were identified by fluorescence. Cell morphology was assessed by acquiring z-stack images and reconstructing three-dimensional models of each cell to be injected. Regions that were distant from the nucleus and from the cell periphery were targeted for injection sites. Pipettes (Femtotips II) were back-filled with an intracellular solution composed of 110 mM KCl, 10 mM NaCl, and 20 mM HEPES (pH 7.2) supplemented with NAADP (100 nM). Cells were injected at a depth of approximately 70% of the cell thickness at the site of injection, with an injection pressure of 85 hPa, a compensation pressure of 40 hPa, a 0.5s injection duration, 45^°^ injection angle, and a 600 μm/s injection speed. For single-channel injection experiments, fluorescence was monitored (λ_ex_ = 488 nm, λ_em_ = 514 ± 15 nm bandpass). Dual-color timelapse imaging was performed by alternating image acquisition of GCaMP6M (λ_ex_ = 488 nm, λ_em_ = 514 ± 15 nm bandpass) and R-CEPIA1er (λ_ex_ = 561 nm, λ_em_ = 590 nm longpass). All injections were performed using a Plan-Apochromat 60×/1.42 oil differential interference contrast objective.

For agonist-evoked Ca^2+^ imaging experiments, cells were cultured in the absence of serum for 16 h prior to imaging, to prevent serum-mediated desensitization of EGF signaling. Cells were washed twice with HBSS with Ca^2+^, and incubated with fluo-4-AM (4 μM), pluronic F127 (0.4%), and probenecid (2.5 mM) for 25 min at room temperature. Cells were then washed twice with HBSS and left at room temperature for 30 min to allow de-esterification. Dishes were mounted on an Olympus IX81 microscope and cells were identified by fluorescence. Fluorescence was monitored (λ_ex_ 488 nm, λ_em_ 514 ± 15 nm bandpass) using a Plan-Apochromat 40×/1.4 oil differential interference contrast objective. For all Ca^2+^ imaging experiments, fluorescence changes were monitored using a Yokogawa spinning disk confocal (CSU-X-M1N), and an Andor iXon Ultra 888 EMCCD camera. Image acquisition and data collection was done using Metamorph version 7.10. Traces of fluorescence intensity over time were plotted and the area under the curve calculated using the curve integration function in Origin (Origin 2020b, OriginLab).

## Data availability

All data necessary for evaluating the conclusions of this study are present within the manuscript and the supporting information.

## Supporting information

This article contains supporting information (23).

## Conflict of interest

The authors declare that they have no known competing financial interests or personal relationships that could have appeared to influence the work reported in this paper.

## References

[1] Lee H.C. (2005). Nicotinic acid adenine dinucleotide phosphate (NAADP)-mediated calcium signaling. J. Biol. Chem..

[2] Morgan A.J., Platt F.M., Lloyd-Evans E., Galione A. (2011). Molecular mechanisms of endolysosomal Ca^2+^ signalling in health and disease. Biochem. J..

[3] Galione A. (2015). A primer of NAADP-mediated Ca(2+) signalling: from sea urchin eggs to mammalian cells. Cell Calcium.

[4] Marchant J.S., Patel S. (2015). Two-pore channels at the intersection of endolysosomal membrane traffic. Biochem. Soc. Trans..

[5] Patel S., Kilpatrick B.S. (2018). Two-pore channels and disease. Biochim. Biophys. Acta Mol. Cell Res..

[6] Gunaratne G.S., Marchant J.S. (2022). The ins and outs of virus trafficking through acidic Ca(2+) stores. Cell Calcium.

[7] Chen C.C., Krogsaeter E., Kuo C.Y., Huang M.C., Chang S.Y., Biel M. (2022). Endolysosomal cation channels point the way towards precision medicine of cancer and infectious diseases. Biomed. Pharmacother..

[8] Marchant J.S., Gunaratne G., Cai X., Slama J.T., Patel S. (2021). NAADP binding proteins find their identity. Trends Biochem. Sci..

[9] Brailoiu E., Hooper R., Cai X., Brailoiu G.C., Keebler M.V., Dun N.J. (2010). An ancestral deuterostome family of two-pore channels mediates nicotinic acid adenine dinucleotide phosphate-dependent calcium release from acidic organelles. J. Biol. Chem..

[10] Rahman T., Cai X., Brailoiu G.C., Abood M.E., Brailoiu E., Patel S. (2014). Two-pore channels provide insight into the evolution of voltage-gated Ca^2+^ and Na^+^ channels. Sci. Signal..

[11] Helle S.C., Kanfer G., Kolar K., Lang A., Michel A.H., Kornmann B. (2013). Organization and function of membrane contact sites. Biochim. Biophys. Acta.

[12] Prinz W.A., Toulmay A., Balla T. (2020). The functional universe of membrane contact sites. Nat. Rev. Mol. Cell Biol..

[13] Kilpatrick B.S., Eden E.R., Hockey L.N., Yates E., Futter C.E., Patel S. (2017). An endosomal NAADP-sensitive two-pore Ca(2+) channel regulates ER-endosome membrane contact sites to control growth factor signaling. Cell Rep..

[14] Cahill M.A. (2007). Progesterone receptor membrane component 1: an integrative review. J. Steroid Biochem. Mol. Biol..

[15] Cahill M.A. (2017). The evolutionary appearance of signaling motifs in PGRMC1. Biosci. Trends.

[16] Kabe Y., Handa H., Suematsu M. (2018). Function and structural regulation of the carbon monoxide (CO)-responsive membrane protein PGRMC1. J. Clin. Biochem. Nutr..

[17] Cahill M.A. (2022). Quo vadis PGRMC? Grand-scale biology in human health and disease. Front. Biosci. (Landmark Ed.).

[18] Cahill M.A. (2022). Unde venisti PGRMC? Grand-scale biology from early Eukaryotes and Eumetazoan animal origins. Front. Biosci. (Landmark Ed.).

[19] McGuire M.R., Espenshade P.J. (2022). PGRMC1: an enigmatic heme-binding protein. Pharmacol. Ther..

[20] Cantonero C., Salido G.M., Rosado J.A., Redondo P.C. (2020). PGRMC1 inhibits progesterone-evoked proliferation and Ca(2+) entry via STIM2 in MDA-MB-231 cells. Int. J. Mol. Sci..

[21] Lee S.K., Kweon Y.C., Lee A.R., Lee Y.Y., Park C.Y. (2022). Metastasis enhancer PGRMC1 boosts store-operated Ca(2+) entry by uncoiling Ca(2+) sensor STIM1 for focal adhesion turnover and actomyosin formation. Cell Rep..

[22] Kabe Y., Nakane T., Koike I., Yamamoto T., Sugiura Y., Harada E. (2016). Haem-dependent dimerization of PGRMC1/Sigma-2 receptor facilitates cancer proliferation and chemoresistance. Nat. Commun..

[23] Lin-Moshier Y., Keebler M.V., Hooper R., Boulware M.J., Liu X., Churamani D. (2014). The Two-pore channel (TPC) interactome unmasks isoform-specific roles for TPCs in endolysosomal morphology and cell pigmentation. Proc. Natl. Acad. Sci. U. S. A..

[24] Gunaratne G.S., Su P., Marchant J.S., Slama J.T., Walseth T.F. (2019). 5-Azido-8-ethynyl-NAADP: a bifunctional, clickable photoaffinity probe for the identification of NAADP receptors. Biochim. Biophys. Acta Mol. Cell Res..

[25] Gunaratne G.S., Brailoiu E., He S., Unterwald E.M., Patel S., Slama J.T. (2021). Essential requirement for JPT2 in NAADP-evoked Ca(2+) signaling. Sci. Signal..

[26] Brailoiu E., Churamani D., Cai X., Schrlau M.G., Brailoiu G.C., Gao X. (2009). Essential requirement for two-pore channel 1 in NAADP-mediated calcium signaling. J. Cell Biol..

[27] Wang-Eckhardt L., Becker I., Eckhardt M. (2021). The PGRMC1 antagonist AG-205 inhibits synthesis of Galactosylceramide and Sulfatide. Cells.

[28] Wang-Eckhardt L., Eckhardt M. (2020). A progesterone receptor membrane component 1 antagonist induces large vesicles independent of progesterone receptor membrane component 1 expression. Biol. Chem..

[29] Ahmed I.S., Rohe H.J., Twist K.E., Mattingly M.N., Craven R.J. (2010). Progesterone receptor membrane component 1 (Pgrmc1): a heme-1 domain protein that promotes tumorigenesis and is inhibited by a small molecule. J. Pharmacol. Exp. Ther..

[30] Yoshitani N., Satou K., Saito K., Suzuki S., Hatanaka H., Seki M. (2005). A structure-based strategy for discovery of small ligands binding to functionally unknown proteins: combination ofin silico screening and surface plasmon resonance measurements. Proteomics.

[31] Eden E.R., White I.J., Tsapara A., Futter C.E. (2010). Membrane contacts between endosomes and ER provide sites for PTP1B-epidermal growth factor receptor interaction. Nat. Cell Biol..

[32] Riad A., Lengyel-Zhand Z., Zeng C., Weng C.C., Lee V.M., Trojanowski J.Q. (2020). The Sigma-2 receptor/TMEM97, PGRMC1, and LDL receptor complex are responsible for the cellular uptake of Abeta42 and its protein aggregates. Mol. Neurobiol..

[33] Riad A., Zeng C., Weng C.C., Winters H., Xu K., Makvandi M. (2018). Sigma-2 receptor/TMEM97 and PGRMC-1 increase the rate of Internalization of LDL by LDL receptor through the formation of a Ternary complex. Sci. Rep..

[34] Furuhata R., Kabe Y., Kanai A., Sugiura Y., Tsugawa H., Sugiyama E. (2020). Progesterone receptor membrane associated component 1 enhances obesity progression in mice by facilitating lipid accumulation in adipocytes. Commun. Biol..

[35] Zhang M., Robitaille M., Showalter A.D., Huang X., Liu Y., Bhattacharjee A. (2014). Progesterone receptor membrane component 1 is a functional part of the glucagon-like peptide-1 (GLP-1) receptor complex in pancreatic beta cells. Mol. Cell. Proteomics.

[36] Hampton K.K., Anderson K., Frazier H., Thibault O., Craven R.J. (2018). Insulin receptor plasma membrane levels increased by the progesterone receptor membrane component 1. Mol. Pharmacol..

[37] Rohe H.J., Ahmed I.S., Twist K.E., Craven R.J. (2009). PGRMC1 (progesterone receptor membrane component 1): a targetable protein with multiple functions in steroid signaling, P450 activation and drug binding. Pharmacol. Ther..

[38] Lee S.R., Kwon S.W., Kaya P., Lee Y.H., Lee J.G., Kim G. (2018). Loss of progesterone receptor membrane component 1 promotes hepatic steatosis via the induced de novo lipogenesis. Sci. Rep..

[39] Knupp J., Chen Y.J., Arunagiri A., Haataja L., Arvan P., Tsai B. (2022). The ER transmembrane protein PGRMC1 recruits misfolded proteins for reticulophagic clearance. Autophagy.

[40] Thejer B.M., Adhikary P.P., Teakel S.L., Fang J., Weston P.A., Gurusinghe S. (2020). PGRMC1 effects on metabolism, genomic mutation and CpG methylation imply crucial roles in animal biology and disease. BMC Mol. Cell Biol..

[41] Thejer B.M., Adhikary P.P., Kaur A., Teakel S.L., Van Oosterum A., Seth I. (2020). PGRMC1 phosphorylation affects cell shape, motility, glycolysis, mitochondrial form and function, and tumor growth. BMC Mol. Cell Biol..

[42] Faris P., Pellavio G., Ferulli F., Di Nezza F., Shekha M., Lim D. (2019). Nicotinic acid adenine dinucleotide phosphate (NAADP) induces intracellular Ca(2+) release through the two-pore channel TPC1 in Metastatic Colorectal cancer cells. Cancers (Basel).

[43] Chen Y.J., Knupp J., Arunagiri A., Haataja L., Arvan P., Tsai B. (2021). PGRMC1 acts as a size-selective cargo receptor to drive ER-phagic clearance of mutant prohormones. Nat. Commun..

[44] Wu H., Voeltz G.K. (2021). Reticulon-3 promotes endosome maturation at ER membrane contact sites. Dev. Cell.

[45] Kwak C., Shin S., Park J.S., Jung M., Nhung T.T.M., Kang M.G. (2020). Contact-ID, a tool for profiling organelle contact sites, reveals regulatory proteins of mitochondrial-associated membrane formation. Proc. Natl. Acad. Sci. U. S. A..

[46] Chambers I.G., Willoughby M.M., Hamza I., Reddi A.R. (2021). One ring to bring them all and in the darkness bind them: the trafficking of heme without deliverers. Biochim. Biophys. Acta Mol. Cell Res..

[47] Donegan R.K., Moore C.M., Hanna D.A., Reddi A.R. (2019). Handling heme: the mechanisms underlying the movement of heme within and between cells. Free Radic. Biol. Med..

[48] Martinez-Guzman O., Willoughby M.M., Saini A., Dietz J.V., Bohovych I., Medlock A.E. (2020). Mitochondrial-nuclear heme trafficking in budding yeast is regulated by GTPases that control mitochondrial dynamics and ER contact sites. J. Cell Sci..

[49] Yuan X., Rietzschel N., Kwon H., Walter Nuno A.B., Hanna D.A., Phillips J.D. (2016). Regulation of intracellular heme trafficking revealed by subcellular reporters. Proc. Natl. Acad. Sci. U. S. A..

[50] Piel R.B., Shiferaw M.T., Vashisht A.A., Marcero J.R., Praissman J.L., Phillips J.D. (2016). A novel role for progesterone receptor membrane component 1 (PGRMC1): a partner and regulator of Ferrochelatase. Biochemistry.

[51] Galmozzi A., Kok B.P., Kim A.S., Montenegro-Burke J.R., Lee J.Y., Spreafico R. (2019). PGRMC2 is an intracellular haem chaperone critical for adipocyte function. Nature.

[52] Aarhus R., Graeff R.M., Dickey D.M., Walseth T.F., Lee H.C. (1995). ADP-ribosyl cyclase and CD38 catalyze the synthesis of a calcium-mobilizing metabolite from NADP. J. Biol. Chem..

