# Supplementary material for: Progesterone receptor membrane component 1 facilitates Ca2+ signal amplification between endosomes and the endoplasmic reticulum
Source: J Biol Chem. 2023 Oct 20;299(12):105378. doi: 10.1016/j.jbc.2023.105378 (PMC10685313; doi:10.1016/j.jbc.2023.105378)
Supplement: Supporting Figures S1–S3 [file mmc1.docx]

**Supplementary Figure 1**

**
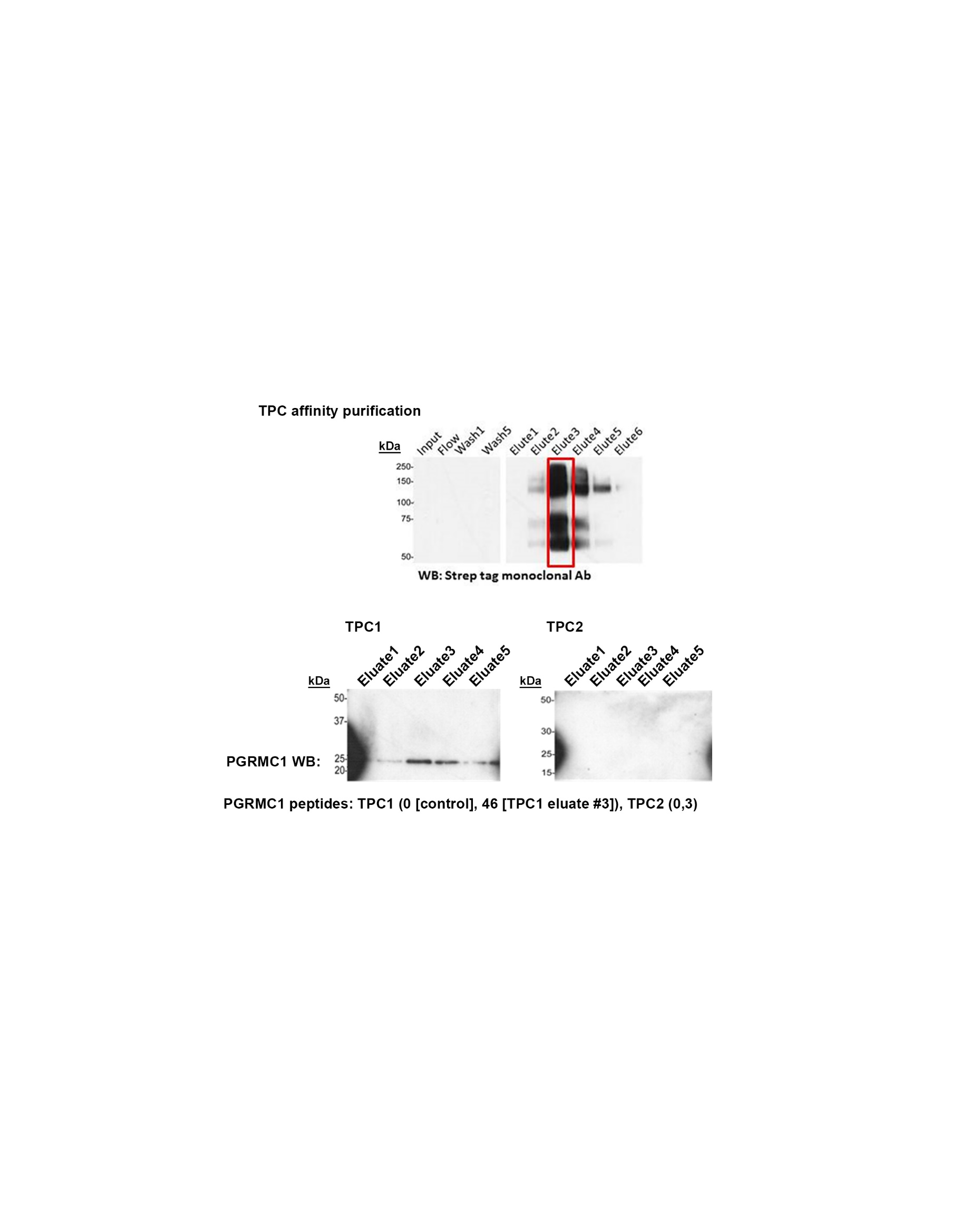
**

**Supplementary Figure 1. Proteomic identification of PGRMC1 as a TPC1 interactor**. *Top*, Representative example showing affinity purification of Strep-tagged TPC isoforms (shown for TPC2) tracked via an anti–Strep-tag monoclonal. Taken with permission from (23). *Bottom*, presence of PGRMC1 in eluate fractions from representation purifications of TPC1 (left) or TPC2 (right). PGRMC1 is preferentially found in TPC1-enriched eluates. Eluate “3” was processed by mass spectrometry and peptide numbers for endogenous PGRMC1 are shown beneath the respective gels.

**Supplementary Figure 2**

**
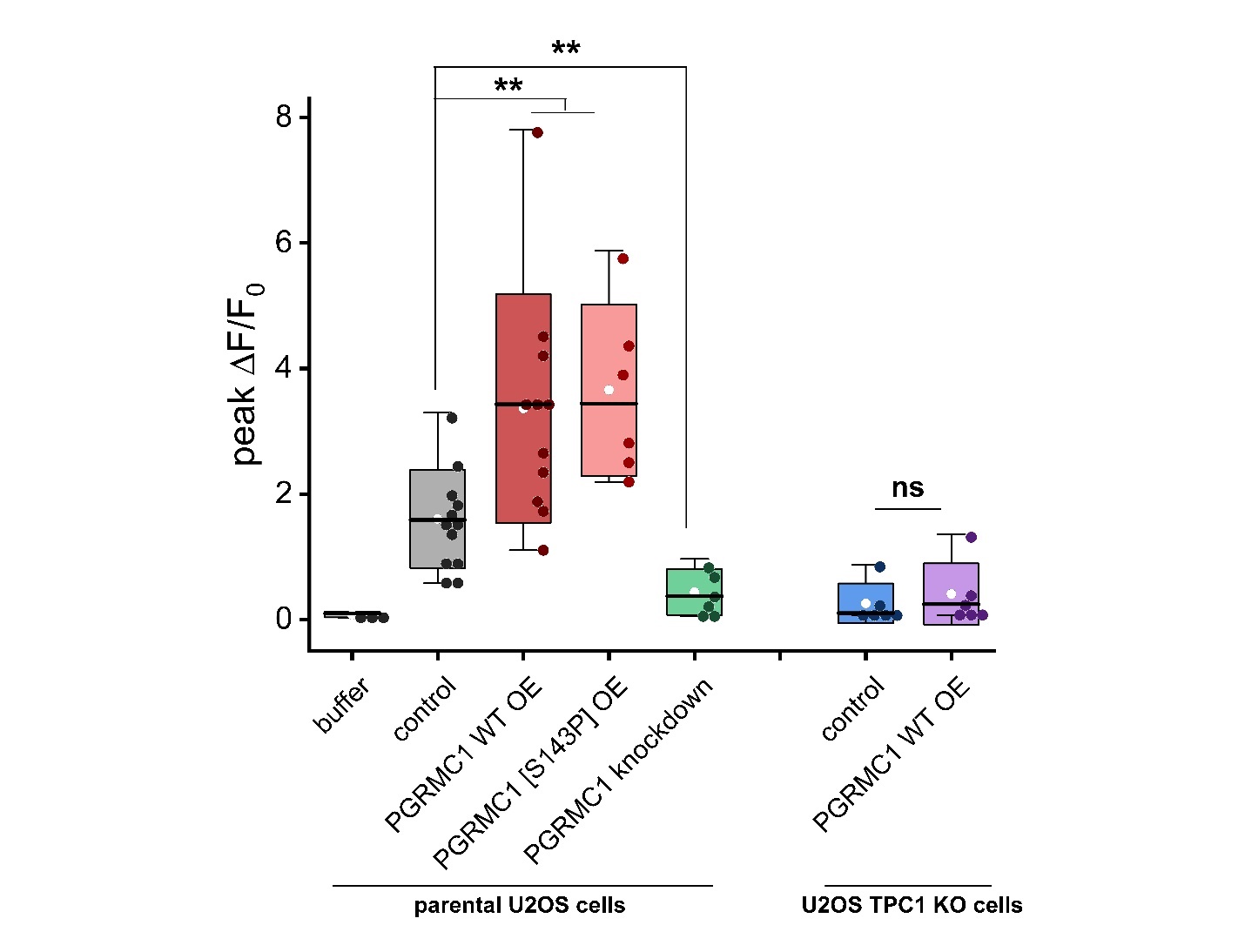
**

**Supplementary Figure 2. PGRMC1 expression levels regulate NAADP signaling in a TPC1-dependent manner.** Peak ΔF/F_0_ values after injection of NAADP (100nM pipette concentration) in parental U2OS cells or U2OS TPC1-knockout cells with transient overexpression of the indicated PGRMC1 variant or transient knockdown of endogenous PGRMC1. Fluorescence changes were detected by GCaMP6M. Data represents mean ± sem values from n ≥ 6 independent injections. Mean (white circles), median (solid line), standard deviation (boxes), 95% confidence interval (whiskers), and independent data points (colored circles) are shown. Statistical significance was determined using paired two-tailed Student’s t test. p-values, *p<0.05, **p<0.01

**Supplementary Figure 3**

**
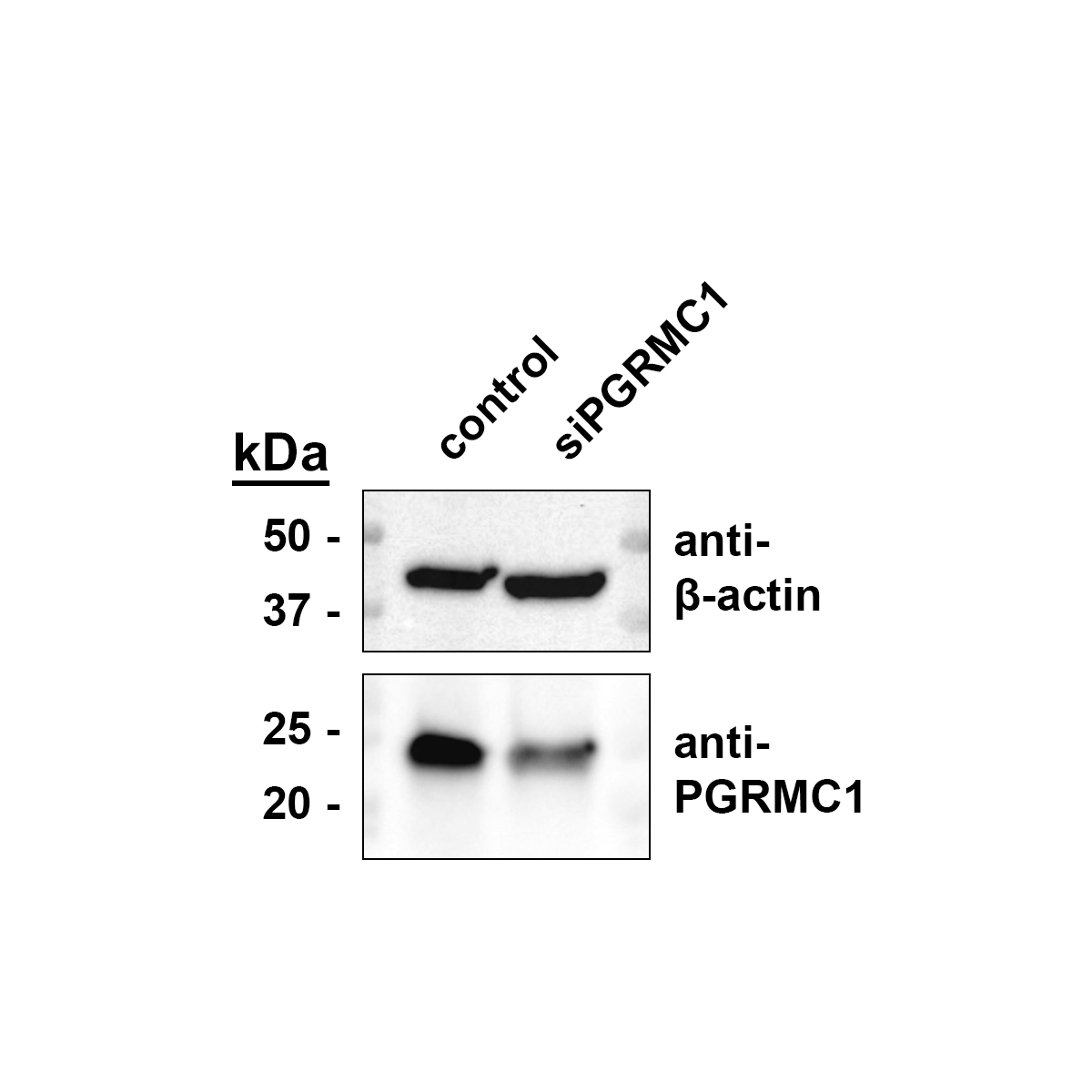
**

**Supplementary Figure 3 – Validation of PGRMC1 siRNA.** Western blot of lysates collected from U2OS cells that were transfected with PGRMC1-specific siRNA.
